# Endometrial pathology in breast cancer patients: Effect of different treatments on ultrasonographic, hysteroscopic and histological findings

**DOI:** 10.3892/ol.2013.1156

**Published:** 2013-01-28

**Authors:** MARIA LE DONNE, ANGELA ALIBRANDI, LEONARDA CIANCIMINO, ANDREA AZZERBONI, BENITO CHIOFALO, ONOFRIO TRIOLO

**Affiliations:** 1 Departments of Gynecological, Obstetrical Sciences and Reproductive Medicine, University of Messina, Messina, Italy; 2 Economical, Business and Environmental Sciences and Quantitative Methods, University of Messina, Messina, Italy

**Keywords:** endometrial carcinoma, tamoxifen, aromatase inhibitors, transvaginal ultrasound, hysteroscopy

## Abstract

Breast cancer patients have an increased risk of endometrial pathology. To investigate whether the incidence of endometrial abnormalities and their clinicopathological features were affected by receiving tamoxifen (TAM), non-steroidal aromatase inhibitors (AIs) or no treatment (NT), 333 peri/postmenopausal breast cancer patients*,* who were referred to the Department of Gynecological, Obstetrical Sciences and Reproductive Medicine for gynecological assessment, were reviewed retrospectively. Transvaginal ultrasonographic (TVUS), hysteroscopic and histological findings were investigated. Endometrial histological findings included: atrophy in 61, 94.3 and 55.6% of cases in the TAM, AIs and NT groups, respectively; polyps in 30.9, 31.4 and 42.2% of cases in the TAM, AIs and NT groups, respectively; hyperplasia in 3% of patients in the TAM group and 11.1% of patients in the NT group; and cancer in 3.8% of cases in the TAM group and 11.1% of cases in the NT group. There was a significant correlation between the duration of TAM treatment and the severity of endometrial pathology. In all groups, there was a significant correlation between hysteroscopic and histological findings with regard to the diagnosis of endometrial atrophy, polyps, hyperplasia and cancer (P<0.001). In conclusion, these data revealed that there was a higher incidence of endometrial pathology in the NT group compared with the TAM group, which was significant for endometrial hyperplasia and cancer. The chance of developing high-risk histological subtypes of endometrial cancer was independent of TAM use. Lastly, although there was no significant difference in recurrent vaginal bleeding and mean endometrial thickness between the TAM and AIs groups, patients receiving AIs did not exhibit hyperplastic, dysplastic or neoplastic changes in the endometrium. This study indicates that breast cancer patients require screening for endometrial pathology; TVUS alone is useful in asymptomatic patients, however, in patients where the endometrial line is irregular or its thickness is >3 mm, hysteroscopy with directed biopsy is the appropriate diagnostic method.

## Introduction

Breast cancer patients have an increased risk of endometrial pathology. The factors involved are not well established, however it may be linked to a hyperestrogenic state and genetic predisposition.

Tamoxifen (TAM) is widely used as an adjuvant hormonal treatment for females with hormone-sensitive breast cancer. It has been effective in reducing the risk of recurrence and mortality via selective estrogen receptor (ER) modulation which causes ER antagonism in breast tissue (1). However, in the endometrium, TAM has ER agonist activity that is able to stimulate proliferation, causing an increased risk of uterine pathologies, including typical and atypical glandular hyperplasia, endome-trial polyps, uterine fibroids, adenocarcinoma and sarcoma (2–9). The risks are highest after long-term use (8,10).

Third generation non-steroidal aromatase inhibitors (AIs), including letrozole and anastrozole, are approved for the treatment of early breast cancer in hormone receptor-positive postmenopausal patients. They decrease estrogen levels by inhibiting the aromatase enzyme, which is responsible for the conversion of androgens to estrogens (11,12). Previous studies have provided evidence which supports the administration of AIs for treating breast cancer, either as 5 years of adjuvant therapy or following 2.5 or 5 years of TAM-based adjuvant therapy as part of a switching strategy (13,14). These had a clinical advantage over TAM treatment (12–14).

Since AIs have no effect on ERs, gynecological symptoms are decreased with AIs treatment compared with TAM treatment; a significant reduction in mean endometrial thickness in patients who switched to anastrozole compared with those who continued on TAM treatment has been observed (13,15–19).

Several approaches have been proposed for screening females with breast cancer for abnormal endometrial proliferation or endometrial cancer. Transvaginal ultra-sonography (TVUS) has improved the measurement and characterization of the endometrium in a variety of clinical situations. However, reported measurements of endometrial thickness of >6–8 mm have subsequently been associated with inactive endometrium following biopsies in TAM-treated patients (20–22). Little benefit has been obtained by the routine use of sonohysterography (23,24). Hysteroscopy with biopsy is the most sensitive and specific method for obtaining a reliable diagnosis, however it is an invasive procedure (9,15,25).

The aim of this study was to investigate whether the incidence of endometrial abnormalities and their clinicopathological features in peri/postmenopausal breast cancer patients, who were referred to the Department of Gynecological, Obstetrical Sciences and Reproductive Medicine for gynecological assessment, were affected by receiving no treatment (NT), TAM or AIs. A further aim of the study was to determine the best approach for screening these patients for endometrial pathology.

## Patients and methods

### Patients

Between January 2002 and December 2011, 333 peri/postmenopausal females, who underwent surgery for invasive breast cancer and had suspected endometrial changes (abnormal vaginal bleeding and/or thickened endometrium), were referred to the Department of Gynecological, Obstetrical Sciences and Reproductive Medicine for gynecological assessment and were reviewed retrospectively. The patients had not received hormone therapy or had gynecological pathology prior to the diagnosis of breast cancer. The study was approved by the Ethics Committee of the University of Messina, Messina, Italy. Written informed consent was obtained from the patients.

### Treatment

TAM was administered to 242 patients (TAM group) at a daily dose of 20 mg, with the exception of two patients who received a daily dose of 40 mg and two patients who received a daily dose of 60 mg. AIs were administered to 36 patients (AIs group) at a daily dose of 2.5 mg and 55 patients did not receive TAM or AIs treatment due to having a hormone receptor-negative status (NT group).

### TVUS evaluation

TVUS was performed using a 5.0-MHz Aloka 500 system. The largest endometrial thickness was recorded on the midline sagittal scan by including the double layer of the endometrium.

### Hysteroscopy

Hysteroscopy was performed by the same operator using a 5, 3 or 1.9 mm, 30-degree optical system (Karl Storz, Tuttningen, Germany) without anesthesia. Sorbitol solution (5% glucose) or 5% mannitol solution was used for uterine distension, with an insufflator that maintained a 100–150-mmHg pressure in the uterine cavity. All patients underwent targeted biopsy during the same procedure and the material was sent for pathological examination.

### Statistical analysis

Numerical variables are expressed as the mean ± standard deviation and the categorical variables are expressed as a number and percentage. A non-parametric approach was used since the examined variables were not normally distributed, as verified by the Kolmogorov-Smirnov test.

Spearman's rank correlation coefficient was applied to evaluate the interdependence between vaginal bleeding and/or treatment duration (in months) and TVUS, hysteroscopic and histological features for each group. The same test was applied to evaluate the overall correlation between TVUS, hysteroscopic and histological findings. The Kruskall-Wallis test was applied to assess the existence of significant differences between the three groups with regard to all clinical, TVUS, hysteroscopic and histological features. The Mann-Whitney U test was applied to perform a two-by-two comparison between the groups.

P<0.05 was considered to indicate a statistically significant difference. The software used for statistical analysis was SPPS for Windows 11.0 (2001; SPSS, Inc., Chicago, IL, USA).

## Results

### General patient data

Only 316 of 333 peri/postmenopausal breast cancer patients were included in this study; 3 patients were withdrawn due to breast cancer recurrence, 6 patients were withdrawn due to hysteroscopy failure as a result of cervical stenosis and in 8 cases, the amount of endometrial material was insufficient for histological diagnosis.

### Clinical features

Clinical features of the study population are shown in Table I. There was no significant difference between the three groups with regard to age, parity, menopausal status and incidence of vaginal bleeding. The age of the patients ranged from 45 to 91 years, with a mean of 59.6 years. All patients were multiparas and 91.1% were postmenopausal.

The mean duration of the TAM and AIs treatment was 41.1 and 33.3 months, respectively.

The mean endometrial thickness, as evaluated by TVUS was 7.7 mm in the TAM group, 6 mm in the AIs group and 4.8 mm in the NT group. Despite no significant difference being identified between the TAM group and the AIs group (P=0.154; Mann-Whitney U test), there was a significant difference between the 3 groups (P=0.009; Kruskall-Wallis test).

### TVUS, hysteroscopic and histological findings

The TVUS, hysteroscopic and histological findings are summarized in Table II. The mean endometrial thickness, as visualized by TVUS, was 5–8 mm in 14.4, 8.6 and 11% of cases and >8 mm in 47, 45.7 and 26.7% of cases in the TAM, AIs and NT groups, respectively; no significant differences were identified between the three groups.

Intracavitary fluid was revealed by TVUS in 11, 2.9 and 6.7% of patients in the TAM, AIs and NT groups, respectively, and there was no significant difference between the three groups. TVUS identified an endometrial lesion in 14, 25.7 and 28.9% of cases in the TAM, AIs and NT groups, respectively.

Hysteroscopy revealed that polyps were present in 38.6, 34.3 and 35.6% of patients and cystic atrophy was concurrently detected in 35.5, 31.4 and 31.1% of patients in the TAM, AIs and NT groups, respectively.

Endometrial atrophy alone was visualized in 13.5% of patients in the TAM group, 62.8% of patients in the AIs group and 44.4% of patients in the NT group. Hyperplastic lesion was diagnosed in 7.2% of cases in the TAM group and 11.1% of cases in the NT group. Neoplastic lesion was suspected in 3% of cases in the TAM group and 8.9% of cases in the NT group.

Endometrial histological findings included: atrophy in 61, 94.3 and 55.6% of cases and polyps in 30.9, 31.4 and 42.2% of cases in the TAM, AIs and NT groups, respectively; hyperplasia in 3% of patients in the TAM group and 11.1% of patients in the NT group; and cancer in 3.8% of cases in the TAM group and 11.1% of cases in the NT group.

One case of cervical carcinoma and one of gastric carcinoma were also diagnosed in the TAM group.

Significant differences were identified between the 3 groups with regard to the incidence of histological diagnosis of atrophy, hyperplasia and endometrial cancer (P<0.001, P=0.015 and P=0.038, respectively). However, hyperplasia, dysplasia and endometrial cancer did not occurr in the AIs group. Furthermore, the incidence of endometrial cancer in the NT group (11.1%) was significantly higher than in the TAM group (3.8%; P=0.038).

Of the 14 endometrial cancer cases observed, although the main histotype was found adenocarcinoma, including endometrioid carcinoma, six cases (42.8%) were high risk histological subtypes, including three papillary serous carcinomas (one case in the TAM group and two in the NT group), two mesodermal mixed tumors in the TAM group and one sarcoma in the NT group.

Amongst patients treated with TAM, there was a significant correlation between the therapy duration and the presence of intracavitary lesion or fluid, as identified by TVUS (P<0.001). No correlation existed between the therapy duration and vaginal bleeding or endometrial thickness.

The length of TAM treatment was also correlated with hysteroscopic identification of suspected cancer (P=0.009) and histological diagnosis of endometrial dysplastic or neoplastic lesions (P=0.009 and P=0.015, respectively). The three documented endometrial cancer histotypes with a poor prognosis in TAM group, one papillary serous carcinoma and two mesodermal mixed tumors, were not correlated with the dosage and occurred after 72, 60 and 160 months of therapy, respectively.

Among patients treated with AIs, there was no significant correlation between therapy duration and vaginal bleeding or TVUS, hysteroscopic and histological findings.

Vaginal bleeding was present in 24.4 and 20% of patients in the TAM and AIs groups, respectively. In the TAM group, vaginal bleeding was significantly correlated with endometrial thickness and intracavitary lesion, as identified by TVUS (P=0.003 and P=0.002, respectively), with hysteroscopically suspected cancer lesion (P=0.003) and with histological diagnosis of endometrial dysplasia (P=0.003). In the AIs group, vaginal bleeding was significantly correlated with hystological diagnosis of endometrial polyp (P=0.047). In the NT group, vaginal bleeding was significantly correlated with intracavitary lesion, as identified by TVUS.

TVUS identification of intracavitary fluid and intracavitary lesion was significantly correlated with hysteroscopic (P=0.013 and P=0.001, respectively) and histological (P=0.049 and P=0.001, respectively) diagnosis of endometrial polyp, as shown in Tables III and IV. There was a significant correlation between hysteroscopic and histological findings, with regard to the diagnosis of endometrial atrophy, polyp, hyperplasia and cancer in all groups (P<0.001), as shown in Table V.

## Discussion

The major systemic treatment for patients with ER^+^ invasive breast cancer is endocrine therapy. Adjuvant therapy with TAM for 5 years reduces the yearly breast cancer mortality rate by 31% (1).

There is conflicting evidence with regard to the association between an increase in endometrial thickness and the duration of TAM treatment (6,10,26,27). In this study, endometrial thickness was not correlated with the duration of TAM treatment. In agreement with other studies, TAM-induced cystically thickened endometrium revealed by ultrasound is not confirmed by hysteroscopy (atrophic endometrium) in 50–90% of cases and has histology which corresponds with condensated stroma and fluid-filled, cystically dilated glands lined with flattened epithelium (28). TAM treatment is associated with an increased risk of developing endometrial cancer; the relative risk is estimated to be two- to six-fold (2,5,29) and this increases with the duration and the cumulative dose of therapy (3,8,10,29,30).

In our study, the presence of endometrial pathology in 41.9% of females treated with TAM confirms, in agreement with the current literature, that long-term TAM use in breast cancer patients is associated with a higher incidence of uterine pathology. Several studies have documented a connection between TAM therapy and benign uterine pathologies (endometrial polyps and hyperplastic endometrial changes). Their incidence exceeds by 3, 9 and 10 times the incidence of endometrial cancer in TAM-treated patients (30), suggesting that the majority of the proliferative effects of TAM on endometrium are unlikely to progress to cancer.

There is no accordance between previous studies with regard to the duration of TAM therapy and the incidence of endometrial pathology. In this study, a significant correlation has been demonstrated between the duration of the TAM treatment and the severity of endometrial pathology.

The process by which TAM contributes to the carcinogenesis of endometrial cancer is unknown; the finding of atypical metaplasia in cystic endometrial atrophy is not explained by estrogenic effects, but points to anti-estrogen or -progesterone-like activity (31).

Bergman *et al*(8) showed that long-term TAM use was positively correlated with p53 overexpression in endometrial tumors and inversely correlated with ER status. p53-positive tumors were often steroid receptor-negative and belonged to a group of malignant mixed mesodermal tumors and endometrial sarcomas (8). Subsequently, Ferguson *et al* observed that gene expression in these tumors was the same as those that have no association with TAM use (32). Although the ER pathway and its role in tumorigenesis in TAM users is controversial, a more frequent expression of ER-β and a lower expression of ER-α was observed by Wilder *et al* in TAM-associated tumors (33).

Weaknesses of our study include its retrospective design and incomplete information about the hormone status (for example, ER^+/−^), histological subtype and stage of breast cancer.

This study showed that the documented endometrial cancer histotypes that had a poor prognosis were not correlated with the dosage or duration of TAM therapy.

Our data were in agreement with those of Slomovitz *et al*, which showed that females who developed endometrial cancer following a diagnosis of breast cancer had an increased risk of developing the high-risk histological subtypes, independent of TAM use. No significant differences were identified in the clinical or pathological features of endometrial cancer between TAM users and non-users (34).

On the other hand, Fles *et al* have demonstrated that tumors (all subtypes) which had developed after prolonged TAM use are not distinguishable from those of non-users on the basis of their genomic profile (35).

Data from this study revealed that there was a higher incidence of endometrial pathology in the NT group than in the TAM group, which was significant for endometrial hyperplasia (11.2 vs. 3%, respectively) and cancer (11.1 vs. 3.8%, respectively). Furthermore, three out of five endometrial cancer cases among TAM non-users were high-risk histological subtypes, including two papillary serous carcinomas and one sarcoma.

These data are in agreement with previous studies which showed that the standardized incidence ratios for uterine cancer in breast cancer patients is increased amongst TAM users and non-users and this may be due to shared etiological risk factors in uterine and breast cancer (36,37).

Patients who were treated for breast cancer had an increased risk of leukemia and gynecological cancer and a slightly enhanced risk of gastrointestinal cancer, in addition to the well-known risk of developing sarcomas and lung cancer after radiotherapy (36–38). In this study, the two mesodermal mixed tumors cases in the TAM group and the sarcoma case in the NT group had undergone radiotherapy.

Adjuvant treatment with AIs has been shown to be more effective than TAM treatment in reducing the risk of recurrence (11–14), with a lower risk of developing endometrial pathology compared with TAM therapy (13–16,19). Furthermore, AIs may reverse TAM-induced endometrial abnormalities (17–20). Treatment with AIs has been shown to significantly reduce the incidence of life-threatening adverse events, including endometrial cancer, compared with TAM treatment (39).

Although no significant differences were identified for recurrent vaginal bleeding and mean endometrial thickness between the TAM and AIs groups, patients in this study who received AIs did not exhibit hyperplastic, dysplastic or neoplastic changes of the endometrium.

Endometrial cancer is a serious adverse event which may occur in breast cancer patients, therefore yearly gynecological assessments and rapid assessment in the occurrence of vaginal bleeding should implemented. TVUS has been reported to be appropriate for screening the endometrium for intrauterine pathology before endocrine treatment in females with breast cancer. An endometrial thickness of 3 mm is the threshold for requiring further investigation with hysteroscopy and endometrial biopsy (15). However, TVUS is not the best method for assessing TAM-induced endometrial changes, due to the high false-positive rate (46–56%) (9,16,22) and there is discordance between TVUS, hysteroscopic and histological findings (9,20,21).

Our data show that TVUS was not accurate in identifying hyperplasia and polyps, as endometrial thickness measurements missed hyperplastic changes and polyps in a large number of cases. Furthermore, for intracavitary lesions, TVUS was unable to differentiate between a polyp, which may contain cancerous cells, and endometrial glandulocystic atrophy. Hysteroscopy is more accurate in the diagnosis of polyps, hyperplastic and neoplastic changes and it is the only method that provides a direct view of the endometrial cavity and the possibility of performing directed biopsies for a definitive diagnosis (9,25).

This study, in accordance with previous studies, indicates that breast cancer patients require endometrial screening, although the cost/efficacy ratio is not favorable. TVUS alone is useful in asymptomatic patients, however, if the endometrial line is irregular or its thickness is >3 mm, hysteroscopy with directed biopsy is the appropriate diagnostic method (9,13,15,40).

## Figures and Tables

**Table I:** Clinical features of the study population.

Variable	TAM	AIs	NT	P-value^a^
Age (years), mean ± SD	59.4±10.6	59.6±11.4	60.6±11.3	ns
Parity, mean ± SD	2.3±1.4	1.9±1.4	2.4±1.9	ns
Therapy duration (months), mean ± SD	41.1±33	33.3±31.4	0	0.000
US endometrial thickness (mm), mean ± SD	7.7±7.2	6.0±7.1	4.8±7.7	0.009
Vaginal bleeding, n (%)	58 (24.6)	7 (20)	14 (31.1)	ns
Menopause, n (%)	216 (91)	32 (91.4)	40 (88.9)	ns

a Kruskall-Wallis test. TAM, tamoxifen; AIs, non-steroidal aromatase inhibitors; NT, no treatment; ns, not significant; US, ultrasonography.

**Table II:** Ultrasonographic, hysteroscopic and histological features.

Variable	TAM, n (%) Total n=236	AIs, n (%) Total n=35	NT, n (%) Total n=45	P-value^a^
Ultrasonography				
5–8 mm	34 (14.4)	3 (8.6)	5 (11.1)	ns
>8 mm	111 (47)	16 (45.7)	12 (26.7)	ns
Intracavitary fluid	26 (11)	1 (2.9)	3 (6.7)	ns
Endometrial lesion	33 (14)	9 (25.7)	13 (28.9)	0.021
Hysteroscopy				
Atrophy	216 (91.5)	34 (97.1)	34 (75.6)	0.002
Polyp	91 (38.6)	12 (34.3)	16 (35.6)	ns
Hyperplasia	17 (7.2)	0	5 (11.1)	ns
Suspected cancer	7 (3)	0	4 (8.9)	ns
Histology				
Atrophy	144 (61)	33 (94.3)	25 (55.6)	<0.001
Polyp	73 (30.9)	11 (31.4)	19 (42.2)	ns
Hyperplasia	7 (3)	0	5 (11.1)	0.015
Dysplasia	5 (2.1)	0	0	ns
Cancer	9 (3.8)	0	5 (11.1)	0.038

a Kruskall-Wallis test. TAM, tamoxifen; AIs, non-steroidal aromatase inhibitors; NT, no treatment; ns, not significant.

**Table III:** Correlation between the ultrasonographic and hysteroscopic findings.

Characteristic	Atrophy	Polyp	Hyperplasia	Suspected cancer
Endometrial thickness	ns	ns	ns	ns
Intracavitary lesion	ns	0.01	ns	ns
Intracavitary fluid	ns	0.013	ns	ns

ns, not significant.

**Table IV:** Correlation between the ultrasonographic and histological findings.

Characteristic	Atrophy	Polyp	Hyperplasia	Dysplasia	Cancer
Endometrial thickness	ns	ns	ns	ns	ns
Intracavitary lesion	0.012	0.001	ns	ns	ns
Intracavitary fluid	ns	0.049	ns	ns	ns

ns, not significant.

**Table V:** Correlation between the hysteroscopic and histological findings.

Variable	Atrophy	Polyp	Hyperplasia	Dysplasia	Cancer
Atrophy	<0.001	ns	<0.001	ns	<0.001
Polyp	ns	<0.001	ns	ns	ns
Hyperplasia	0.02	ns	<0.001	ns	ns
Suspected cancer	<0.001	0.019	ns	0.042	<0.001

ns, not significant.
